# Editorial

**Published:** 1899-03

**Authors:** 


### Editorial.

### SEMI-CENTENNIAL MEETING OF THE MEDICAL ASSOCIATION OF GEORGIA IN MACON.

The next meeting of the State Medical Association will commemorate the fiftieth anniversary of the founding of the society. The meeting gives promise of being the largest and most attractive ever held. The following is taken from a letter received from Dr. Howard J. Williams, the very active and efficient President of the Association:

"The meetings will be held in the Academy of Music, April 19, 20 and 21. The hall has a capacity to seat comfortably all the doctors of Georgia if they will come, and has very convenient committee-rooms, writing-rooms, lavatories, telegraph office, stenographers, etc. The stage will be decorated with flowers and the portraits of Dr. Lewis D. Ford (first president) and Dr. Crawford W. Long (a first member and the discoverer of anæsthesia in surgical practice).

"The address of welcome will be delivered by the Hon. Emory Speer, Judge of the United States Court and an orator of the first rank. The reply to the address of welcome will be by Dr. DeSaussure Ford (a former president and son of the first president). The five living first members, J. F. Alexander, F. M. Brantly, W. L. Jones, W. D. Maddox and C. F. Redding, will be the guests of honor of the Association and will occupy seats on the stage and be otherwise honored during their stay in Macon.

"Dr. Nicholas Senn, of Chicago, will be present and will read a paper on 'Emergency Surgery.'

"Dr. Hobart A. Hare, of Philadelphia, will also attend the meeting and present a paper on 'Facts Relative to the Medical Complications, Sequellæ and Treatment of Typhoid Fever.'

"Dr. Joseph Price, of Philadelphia, will come and read a paper on 'An Analysis of Appendicitis Operation Methods.'

"These papers will be discussed thoroughly by good men—members of the Association, which, of course, will add to their value.

"A symposium of ether anæsthesia in honor of the memory of Crawford W. Long will be taken up by four leading members of the Association, and discusssed by others. The members of the Association will wear unique souvenir buttons bearing pictures of Dr. Ford and Dr. Long.

"The committee on new members are at work, and their chairman reports that there will be an increase of membership this year far in advance of any previous year. We thought in 1897 when at the Macon meeting we took in 104 new members that we had reached the high-water mark, but it is to be a flood this year. The committee of arrangements is at work to give the Association a grand time. They are assisted by the local medical society and the citizens generally. A grand reception for the first evening (19th) and a great entertainment the second evening are in preparation, while private dinners, luncheons and breakfasts will be added to make everybody have a good time. Exhibitors of medical supplies, surgical instruments and medical books are clamoring for space, so that department will be of the first class.

"Our hotels, seven in number, will give special association rates, ranging from one to two and a half dollars per day, according to the hotel, for visiting doctors and their families. A list of private boarding-houses will be provided for those who can not secure accommodations at the hotels. The railroads will give specially reduced rates this year—lower than for any previous meeting.

"We are looking for a big crowd and are getting ready to entertain them. We put the probable attendance at 500.

"In the way of scientific work the program committee is making every effort to keep up with the other committees, so that in addition to the contribution of the visiting medical gentlemen I have mentioned, we shall have a grand program."

As most of the members of the Association know of Dr. Williams' ability to promote the success of a meeting, it need scarcely be impressed upon them that the rose-colored view of the situation expressed in his letter is warranted by facts.

We urge each member to "lay aside all other business" and attend the Macon meeting in April. The strength of the Association depends upon the interest taken in it by its members. Embody all you know and have observed of a subject interesting to you in the form of a paper and present it in person at the meeting. If it interests you, it is sure to interest others, and after reading it, you will go home with the comfortable feeling] that you were a factor in the success of the meeting. Urge your medical friends to come to the meeting with you and join the Association. Every regular practitioner in the State ought to be a member, and there is no justifiable excuse for his not joining at the coming meeting. One attendance will convince him of the value of membership not only as to his work, but to his professional standing as well.

Although the list of papers to be presented will be large, every man, so far as possible, will be given a chance to read his. At any rate it will appear in the volume of the transactions, of which each member is entitled to a copy. If you desire to present a paper, it would be well to read over Art. VIII. of the by-laws, which is as follows:

PAPERS.

SEC. 1. The titles of all papers to be read at any annual meeting shall be furnished the chairman of the program committee (Dr. R. B. Barron, Macon, Ga.) thirty days before the meeting.

SEC. 2. Not more than twenty minutes shall be occupied in reading any paper. No speaker shall be allowed to speak a second time on the same subject nor to consume more than five minutes in the discussion.

SEC. 3. No paper shall occupy over twenty pages of the transactions.

SEC. 4. The publication committee shall decline to publish any paper not handed to the secretary complete before the final adjournment of the annual meeting.

SEC. 5. No paper shall be read before the Association which has already been published or has been read before any other body.

SEC. 6. A member presenting a paper must be present at the meeting in order to have it published in the transactions.

---

Dr. George B. Stone, of Savannah, is dead.

---

Dr. Geo. T. Simmons, of Lincoln, Nebraska, has been appointed editor of the Journal of the American Medical Association, to occupy the vacancy created by the death of Dr. John B. Hamilton. Will some one please rise and explain who Dr. Simmons is?

---

We are pleased to state that although their building at 1228 Market street was completely destroyed by fire, Messrs. Wm. R. Warner & Co. are now located at No. 639 N. Broad street, Philadelphia, in their new laboratory and are filling orders as though nothing had happened. This is another demonstration of the invincible American spirit that will rebound in the very face of the bitterest disaster. We are all glad to learn of their rapid recuperation. Warner & Co. have always been the staunch friends and allies of the doctor, and any misfortune which might overtake them would be a source of regret to him.

---

Professor Rontgen has declined to accept the chair offered him at the University of Leipzig, preferring to remain with the University of Wurzburg.

---

Dr. C. I. Taylor, well known as an orthopedic surgeon, recently died at Los Angeles, Cal.

